# Unifying NHS Electronic Prescribing and Health Records: Bridging the Digital Divide

**DOI:** 10.7759/cureus.86055

**Published:** 2025-06-15

**Authors:** Hassan Ahmed, Syeda Aiman Rizvi

**Affiliations:** 1 Internal Medicine, Queen Elizabeth Hospital, King's Lynn, GBR; 2 Internal Medicine, Karachi Medical and Dental College, Karachi, PAK

**Keywords:** economic efficiency, electronic health record (ehr), national health services, nhs, unified health system

## Abstract

The NHS is facing significant challenges in delivering safe, efficient, and high-quality care due to the fragmentation of patient information. A unified electronic health record (EHR) is essential to address several emerging issues, improve patient safety, and deliver better healthcare. Currently, healthcare professionals must navigate multiple systems to access vital patient data, which can lead to ambiguity, miscommunication, errors, and delays in healthcare provision. A single integrated system will enable real-time access to patient records, preventing misdiagnosis, reducing errors, and treatment delays, ultimately resulting in enhanced patient outcomes. Financially, a unified system would reduce software costs and duplicate investigations, resulting in cost savings that can be reinvested in other areas of healthcare. Moreover, the system would benefit medical research by enabling access to comprehensive patient data for tracking healthcare trends and focusing more on evidence-based practice. The NHS’s centralized structure positions it well for system-wide implementation. Regional pilot projects demonstrate feasibility and offer a roadmap for national rollout. While challenges remain, including funding, training, and data privacy, the long-term benefits far outweigh them. A unified EHR system is a strategic necessity for a safer, more efficient, and equitable NHS.

## Editorial

I am writing to highlight the pressing need for a unified electronic prescribing and health information system across the NHS. The current fragmentation of patient records, reliant on disconnected and often incompatible platforms, creates inefficiencies and compromises patient safety and care continuity [1]. A single, integrated system would not only address these shortcomings but also deliver significant benefits in safety, efficiency, research, and cost-effectiveness. 

One of the most critical advantages of a unified system is the improvement of patient safety. At present, healthcare professionals must navigate multiple systems to access essential data, including blood test results, imaging, medications, and discharge summaries. This fragmentation leads to delays and increases the risk of errors. A single platform offering real-time access to patient records would reduce these risks, enabling clinicians to make faster, more informed decisions and minimizing adverse drug interactions, prescription errors, and misdiagnoses. 

Beyond safety, such a system would greatly enhance efficiency. Clinicians frequently lose valuable time switching between disparate systems, attempting to compile a comprehensive patient history. A centralized platform would streamline workflows by consolidating all relevant information in one place and reducing administrative burdens. This would allow healthcare professionals to dedicate more time to patient care rather than navigating complex IT systems. 

A fully integrated system would also facilitate seamless coordination across healthcare settings, including hospitals, GP practices, ambulance services, and allied health professionals such as physiotherapists and occupational therapists. At present, patient records do not always transfer smoothly between care providers, leading to gaps in information, delays, and the potential for miscommunication. A unified electronic health record (EHR) system would ensure all providers have access to the same up-to-date patient information, thereby improving care continuity and reducing the risk of treatment errors. 

The NHS is uniquely positioned to implement such a system. Unlike more fragmented healthcare systems worldwide, the NHS operates under a single-payer model, allowing for standardized data management across all care settings. This structural advantage makes large-scale integration feasible and more straightforward than in other healthcare environments. 

Financially, the benefits of a unified system are substantial. Currently, numerous NHS trusts subscribe to different clinical applications, resulting in duplicated costs, fragmented data, and inefficiencies. A single, standardized platform would eliminate the need for multiple software subscriptions, reduce unnecessary diagnostic tests, and cut administrative overhead. These savings could be reinvested in frontline care, bolstering NHS resources and improving patient outcomes [2]. A self-developed, home-grown system would be the most cost-effective and sustainable approach in the long term. By building an in-house platform, the NHS could avoid expensive licensing fees and maintain full control over system updates, security, and functionality. This approach would also allow for future adaptability, ensuring that the system evolves in response to emerging healthcare needs. 

A unified EHR system would also be an invaluable asset for medical research and clinical audits. Anonymized, aggregated patient data could facilitate the tracking of health trends, evaluation of treatment outcomes, and continuous quality improvement [3]. Access to large-scale datasets would enhance evidence-based practice, ensuring that healthcare delivery remains aligned with the latest medical advancements. 

Furthermore, the system could provide critical insights into healthcare inequalities. By analyzing patient data across demographics, the NHS would be better equipped to identify disparities in care delivery and outcomes, enabling targeted interventions to reduce health inequities. This would support efforts to ensure equitable access to high-quality care for all patient populations. 

Encouragingly, progress toward such a system is already underway. In the East of England, three NHS trusts are collaborating to implement a unified electronic patient record (EPR) system across their organizations [4]. This initiative could serve as a blueprint for national expansion, demonstrating the tangible benefits of an integrated, standardized platform. The new EHR should be built from the ground up, considering the frequent cyber attacks [5]. If successfully implemented at scale, this approach could revolutionize the NHS, setting a precedent for a fully unified digital health infrastructure. 

A unified Electronic Patient Record (EPR) system would transform the NHS by enhancing patient safety through reduced medication errors and improved diagnostic accuracy; improving care coordination by enabling seamless information sharing among healthcare providers; advancing research through access to anonymized data for clinical trials and public health studies; and increasing efficiency by automating administrative tasks and reducing paperwork, ultimately addressing healthcare inequalities and lowering overall costs. For instance, remote monitoring of patients with chronic conditions, facilitated by a unified system, can improve outcomes and reduce the need for expensive hospital visits. Digital transformation is a necessity, not a luxury, for a modern, effective, and equitable healthcare service capable of meeting the evolving needs of the population. Decisive action, including dedicated funding, clear timelines, and strong leadership, is needed to realize this vision and create a truly integrated and digitally enabled NHS. 

A nationwide electronic prescribing and health information system, including EHRs and e-prescribing, is crucial for the NHS, enabling seamless data sharing and improved decisions. While implementation faces challenges like costs, training, and General Data Protection Regulation (GDPR) compliance, the long-term benefits outweigh these hurdles. The initial investment is offset by savings from reduced errors, fewer readmissions, and streamlined processes.
